# Gestational Diabetes Mellitus and the Long-Term Risk for Glucose Intolerance and Overweight in the Offspring: A Narrative Review

**DOI:** 10.3390/jcm9020599

**Published:** 2020-02-22

**Authors:** Hannah Nijs, Katrien Benhalima

**Affiliations:** 1 Medical school, University Hospital Gasthuisberg, KU Leuven, Herestraat 49, 3000 Leuven, Belgium; hannah.nijs@student.kuleuven.be; 2 Department of Endocrinology, University Hospital Gasthuisberg, KU Leuven, Herestraat 49, 3000 Leuven, Belgium

**Keywords:** gestational diabetes mellitus, long-term metabolic outcome, offspring, overweight, obesity, adiposity, glucose intolerance, abnormal glucose tolerance, insulin resistance

## Abstract

Gestational diabetes mellitus (GDM) is a common condition with increasing prevalence worldwide. GDM is associated with an increased risk for maternal and neonatal complications. In this review we provide an overview of the most recent evidence on the long-term metabolic risk associated with GDM in the offspring. We conducted an extensive literature search on PubMed and Embase between February 2019 and December 2019. We performed a narrative review including 20 cohort studies, one cross-sectional study, and two randomized controlled trials. Our review shows that the prevalence of overweight/obesity and glucose intolerance is higher in children exposed to GDM compared to unexposed children. Maternal overweight is an important confounding factor, but recent studies show that in general the association remains significant after correction for maternal overweight. There is limited evidence suggesting that the association between GDM and adverse metabolic profile in the offspring becomes more significant with increasing offspring age and is also more pronounced in female offspring than in male offspring. More research is needed to evaluate whether treatment of GDM can prevent the long-term metabolic complications in the offspring.

## 1. Introduction

Gestational diabetes mellitus (GDM) is a worldwide public health problem. The prevalence is increasing due to delayed motherhood, the rising prevalence of obesity, and unhealthy lifestyles. The prevalence of GDM ranges from 1.8–31.5%, depending on the used diagnostic criteria and the population studied [1]. Since glucose crosses the placenta, GDM leads to fetal hyperglycemia, which in turn causes hyperinsulinemia. Since insulin acts as a growth hormone during pregnancy, this will induce macrosomia-related perinatal adverse outcomes [2]. In recent years, there is increasing evidence that intrauterine exposure to hyperglycemia also influences the long-term outcome of the offspring [3]. Many studies have shown that GDM increases the risk of glucose intolerance and overweight in the offspring [4]. However, it is less clear whether these associations are based on a direct relationship or are mediated by confounding factors such as maternal obesity. The use of different diagnostic criteria for GDM and the fact that not all studies have corrected for potential confounding factors, could partially explain the inconsistent results. In addition, the susceptibility to a potential effect of GDM may vary by age and gender of offspring. Further clarification is needed, since the prevalence of overweight and obesity in children is increasing [5,6]. These children are likely to become obese as adults and have an increased risk for diabetes and cardiovascular diseases [6,7,8]. It is therefore important to evaluate whether GDM is an independent risk factor and whether treatment of GDM can reduce the long-term metabolic risk in the offspring. We performed a review to evaluate whether GDM is an independent risk factor for glucose intolerance and overweight in the offspring. We included therefore studies evaluating the long-term metabolic risk in offspring from mothers with GDM compared to offspring of mothers with normal glucose tolerance. In addition, we determined whether the risk varied according to gender and age of the offspring.

## 2. Methods

### 2.1. Data Sources and Search Strategies

Between February 2019 and December 2019, a literature search was conducted on PubMed and Embase. We included studies published from 2000 onward. Cross-sectional studies, case-control studies, cohort studies, and randomized controlled trials (RCT) were considered for this review. This is a narrative review. We did not perform a systematic review and could therefore not perform a meta-analysis.

We used the following inclusion criteria:

1. The study population were offspring born to mothers with GDM (OGDM).
2. The control group could either be offspring of mothers with normal glucose tolerance (NGDM) or offspring of mothers with intensive (with insulin or other pharmacological treatment) treated GDM.
3. The following comparisons were made: the OGDM group was compared to the NGDM group or children of mothers with untreated GDM were compared to children of mothers with intensive treated GDM.
4. The different outcomes studied related to adiposity were overweight and obesity (defined by sex- and age-specific reference values according to the International Obesity Task Force, Centers of Disease Control and Prevention, World Health Organization, or local criteria), body fat percentage (BF%), waist circumference (WC), and body mass index (BMI). The outcomes studied related to glucose intolerance were abnormal glucose tolerance (AGT) and indices of insulin sensitivity and beta-cell function. AGT was defined as pre-diabetes or type 2 diabetes mellitus (T2DM). Pre-diabetes was defined as the presence of impaired fasting glucose (IFG) and/or impaired glucose tolerance (IGT). Insulin sensitivity was defined using the Matsuda index, a measurement of whole-body insulin sensitivity [9] or homeostatic model assessment of insulin sensitivity (HOMA-S), a measure of largely hepatic insulin sensitivity [10]. HOMA-S is defined as the reciprocal of insulin resistance (1/HOMA-IR) [10]. As measures of beta-cell function, the insulinogenic index and the disposition index (DI), were used [11,12]. DI was calculated by combining measurements of insulin secretion and sensitivity according to different formulas used in the included articles.

We excluded animal studies, descriptive designs (case series and case reports), studies that made no distinction between the different types of diabetes, studies with a low quality (no method section, no *p*-values mentioned, less than 100 participants), and articles written in a language other than English or French. We did not limit our search to a specific population or ethnicity or to a specific age category. We used the following search strategies:

1. PubMed: (“Diabetes, Gestational”[Mesh]) AND (“Child, Preschool”[Mesh] OR “Child”[Mesh] OR “Adolescent”[Mesh] OR “Adult Children”[Mesh]) AND ((“Diabetes Mellitus, Type 2”[Mesh] OR “Blood Glucose”[Mesh] OR “Insulin/blood”[Mesh] OR “Insulin Resistance”[Mesh] OR “Hyperglycemia/blood”[Mesh] OR “Glucose Intolerance”[Mesh] OR “Prediabetic State”[Mesh]) OR (“Adiposity”[Mesh] OR “Body Mass Index”[Mesh] OR “Obesity”[Mesh] OR “Overweight”[Mesh]))
2. Embase: ‘pregnancy diabetes mellitus’/exp AND ‘progeny’/exp AND (‘obesity’/exp OR ‘body mass’/exp OR ‘non insulin dependent diabetes mellitus’/exp OR ‘glucose intolerance’/exp OR ‘glucose blood level’/exp OR ‘hyperglycaemia’/exp OR ‘insulin resistance’/exp OR ‘impaired glucose tolerance’/exp). We limited our search results by using the mapping option “Limit to terms indexed in article as major focus”.

In addition to this, we hand-searched the reference lists of the selected articles and relevant reviews.

### 2.2. Data Synthesis and Analysis

The extracted data included the study design, location, age of follow-up, number of study participants, the GDM diagnosis criteria, adjustments that were made, and offspring outcomes. We reported our results in a descriptive manner. A *p*-value <0.05 was considered to be significant.

## 3. Results

### 3.1. Search Results

**Figure 1:**
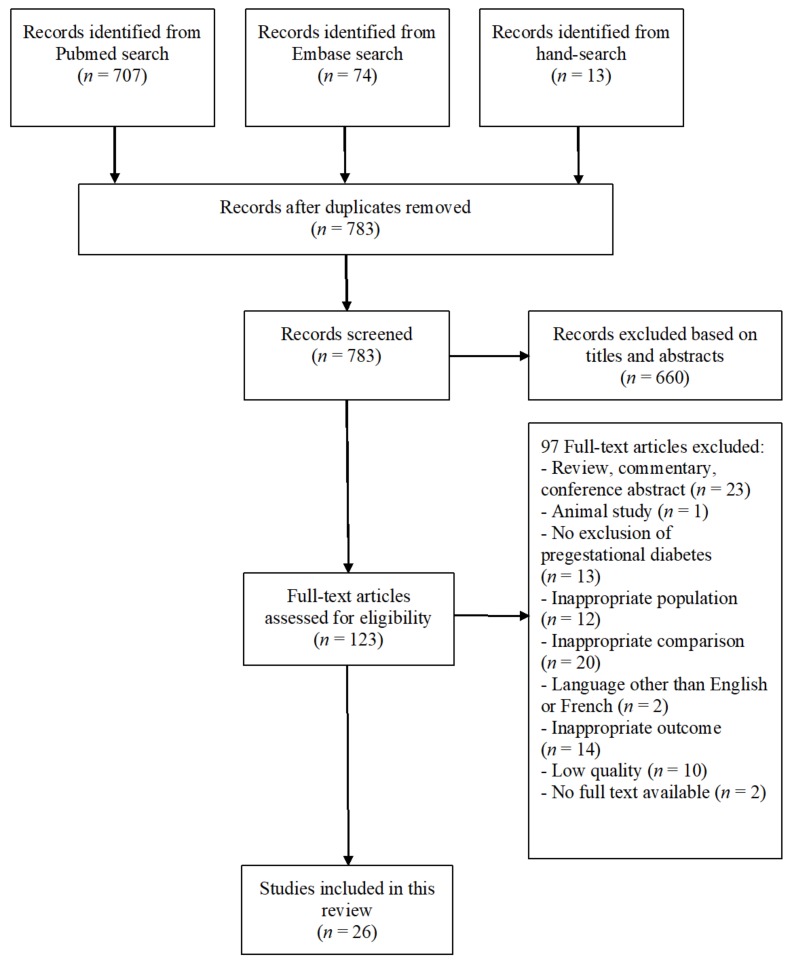
The literature search and selection process.

We identified 783 articles of which 123 articles were selected as possibly relevant. After examination of the full text, 23 studies were included in the current review (Figure 1).

### 3.2. Study Characteristics

The study characteristics are shown in Table 1. In total, there were 15 prospective cohorts (65%), five retrospective cohorts (22%), one cross-sectional study (4%), and two RCTs (9%). Three studies were performed in Asia (13%), six studies in North America (26%), 11 in Europe (48%), one was performed in Oceania (4%), and two studies were multinational (9%). All 23 studies were published between 2003 and 2019, of which 19 studies (83%) were published from 2010 onward. The follow-up ranged from one to 27 years. In 13 studies (57%), the offspring was older than ten years. Only three studies (13%) evaluated the impact on adult offspring (>18 years). The sample size varied between 129 and 14,881 participants. Eighteen studies (78%) evaluated more than 500 children. Two studies used the ‘International Association of Diabetes and Pregnancy Study Group’ (IADPSG) criteria (9%), five studies used the Coustan and Carpenter criteria (22%), two studies the American Diabetes Association criteria (9%), three studies the Finnish Diabetes Association criteria (13%), one study the German Diabetes Association criteria (4%), three studies used local standards (13%), and three studies used multiple criteria for GDM (13%). In four studies (17%), no information was available about the diagnostic criteria used for GDM. Appendix A Table A1 gives an overview of the most commonly used diagnostic criteria for GDM across the different studies.

### 3.3. Overweight and Obesity

The prevalence of overweight and obesity was higher in children exposed to GDM compared to the control group (Table 2). In the OGDM group, 21–40% of the children were overweight (including obesity) compared to 10.4–30% in the NGDM group, and 6.4–20.2% of the children were obese compared to 1.9–12.2% in the NGDM group.

Nine studies reported an odds ratio (OR) for overweight. Four studies showed a significantly increased OR of 1.44–2.29 for overweight in the OGDM group [16,19,23,33]. These four studies adjusted for maternal BMI, but after this adjustment, the result remained significant in only two studies [23,33]. Pirkola et al. showed a higher risk of overweight in the offspring of overweight GDM mothers, but not in the offspring of normal-weight mothers [29]. In contrast, Grunnet et al. demonstrated a significant higher BMI in the offspring of normal-weight GDM mothers (mean difference 5%, 95% CI 3–7%) but not in the offspring of underweight or overweight GDM mothers [18]. Hillier et al. demonstrated a significant increased OR of overweight in the offspring of mothers diagnosed with GDM based on the Carpenter and Coustan criteria (untreated women) but not in the offspring of mothers diagnosed with GDM based on the National Diabetes Data Group (treated women) criteria [35].

Five studies reported an OR for obesity. Four showed a significantly increased OR of 1.53–3.59 in the OGDM group. Three of these studies adjusted for maternal BMI and after adjustment the result remained significant in two studies [16,23]. Hillier et al. only showed a significant increased OR in the offspring of untreated women [35].

Seven studies evaluated the impact of GDM on the waist circumference. Four of them showed a significantly increased waist circumference in the OGDM group [16,21,23,34], of which three studies corrected for maternal BMI [16,21,23].

The Hyperglycemia and Adverse Pregnancy Outcome (HAPO) follow-up study evaluated 4832 children of untreated women (defined post hoc by the 2013 WHO criteria) 10–14 years after delivery. This study showed a continuous association between maternal glucose levels during pregnancy and a higher risk of adiposity [13]. Each standard deviation (SD) increase in maternal fasting plasma glucose (FPG) was associated with an increased risk of obesity and body fat percentage >85th percentile, but not with an increased risk of overweight or a high waist circumference. A higher maternal plasma glycemia level 60 and 120 min after an OGTT was related to an increased risk of all these adiposity outcomes. These results were independent of maternal BMI.

### 3.4. Glucose Intolerance

We included seven studies that investigated the impact of GDM on AGT and insulin resistance (IR) in the offspring (Table 3). Four of these studies did not correct for any confounding factor. Five studies evaluated AGT as a whole (IFG and/or IGT and/or T2DM). Three of these demonstrated an increased incidence of AGT in the OGDM group, with a total AGT prevalence of 21–41% in the OGDM group compared to 4–15.3% in the NGDM group [12,33,34]. This relationship seems linear related to rising glycemic values in pregnancy since Tam et al. demonstrated an OR for offspring’s AGT of 1.85–2.00 for each SD increase in maternal glycemic level (adjusted for confounding factors including maternal weight and neonatal weight) [19].

Five studies investigated FPG separately. Only one study showed a significant higher FPG level in the OGDM group compared to the NGDM group (mean difference 4%, 95% CI 2–5%) [18]. In addition, the HAPO follow-up study showed an increased risk for IGT (OR 1.96, 95% CI 1.41–2.73) in the OGDM group and demonstrated that this increased risk was linear across the spectrum of maternal glucose levels during pregnancy [14,15]. This association was independent of maternal BMI, child BMI, and child’s family history of diabetes.

Five studies evaluated indices of IR in the offspring. Four studies showed an increased IR in the OGDM group, defined by HOMA or the Matsuda index [12,14,18,34]. GDM was not associated with a decreased insulinogenic index in the offspring, although there was a significant decreased DI in the OGDM group [12,14,19]. The HAPO follow-up study showed an inverse continuous relationship between maternal pregnancy glucose levels and child insulin sensitivity and DI [15]. This association was attenuated, but remained significant, after adjustment for maternal BMI and/or child BMI

### 3.5. Age

Only two studies examined different age categories. One study observed an increased incidence of overweight in 8- and 11-year-old OGDM but not in 2-year-old OGDM, while the other study showed an increased HOMA-IR in 9.5-year-old OGDM but not in 5-year-old OGDM [27,31]. Of all five studies evaluating young children, only one study showed an increased risk of overweight or obesity in OGDM <10 years. In contrast, of all nine studies evaluating older children, five studies showed an increased risk of overweight or obesity in OGDM ≥10 years. Only one study examined the impact on glucose intolerance in offspring <10 years and showed a significant increased risk of AGT in the OGDM group [19].

### 3.6. Sex Differences

Of all studies, only seven studies evaluated possible sex differences. Four studies showed a significant increase in adiposity measures in 7–25-year-old girls, but not in boys, when exposed to intrauterine GDM. Girls from mothers with GDM had a significantly higher prevalence of overweight (22.7% vs. 13.0%, *p* = 0.03) [19], higher BMI (16.4 kg/m^2^ vs. 14.3 kg/m^2^, *p* <0.001) [31], and higher waist circumference [8,21]. In contrast, only one study showed an OR for overweight of 2.34 (95% CI 1.26–4.34) in 5–7-year-old male OGDM and no increased risk in females [17]. Another study showed no difference in both waist circumference and BMI, irrespective of gender [24]. Only one study examined the differences in sex with regard to glucose intolerance and insulin resistance [31]. This study showed a significant increased IR and plasma glucose levels 30 min after OGTT in 9.5-year-old female OGDM but not in males.

### 3.7. Can Treatment of GDM Reduce the Long-Term Metabolic Complications in the Offspring?

Two RCT’s have shown that treatment of GDM lowers the risk of perinatal adverse outcomes [37,38]. The ‘Australian Carbohydrate Intolerance Study in Pregnant Women’ (ACHOIS) was an RCT in women with GDM based on the 1999 WHO criteria [38]. The Landon RCT was a multicenter study that randomly assigned 958 American woman who met the criteria for mild GDM (FPG <5.3 mmoL/L; two abnormal values after a 100 g OGTT according to the Carpenter and Coustan criteria) into a treatment group and a control group and compared the infants of both groups [37]. The follow-up study of ACHOIS was conducted in 4–5-year-old offspring of women who lived in the state of South Australia and were checked by a health care program at kindergartens and preschools (resulting in 199 children, 19% of the original cohort) [30]. Treatment of GDM did not result in a change in BMI. The Landon follow-up study evaluated the treatment effect of GDM on BMI in 500 children (52% of the original cohort), aged 5–10 years [22]. Treatment was also not associated with a reduction in childhood obesity. However, a subanalysis showed higher rates of IFG in girls of mothers in the control group compared to the treatment group (12.1% vs. 2.9%, *p* = 0.02). In addition, treatment of GDM was associated with a decreased frequency of HOMA-IR in female offspring (1.05 vs. 1.30, *p* = 0.04). These results were adjusted for race/ethnicity and maternal baseline BMI.

## 4. Discussion

### 4.1. Summary of Findings

In the current review, we show that there is increasing evidence that offspring of mothers with GDM are at increased risk for overweight and glucose intolerance and that this risk is independent of maternal overweight. In addition, recent studies, such as the HAPO follow-up study, also demonstrated a linear relationship between rising maternal glucose levels during pregnancy and the risk for overweight and AGT in the offspring.

### 4.2. Results in Relation to What We Already Know

In recent years, there is increasing evidence that GDM is associated with an increased risk for overweight and AGT in the offspring [4]. However, it remained less clear whether these associations are based on a direct relationship or whether they are mediated by confounding factors. Maternal overweight is a well-known confounding factor, likely due to shared genes and environment [39]. In this review, we aimed to investigate whether GDM is a risk factor independent of maternal BMI.

We showed that the association between GDM and offspring overweight or obesity is frequently attenuated after adjustment for maternal BMI, but that the association remained significant in recent, large studies. The HAPO follow-up study even showed a continuous relationship between maternal glycemia levels during pregnancy and childhood adiposity outcomes [14]. This study was not confounded by treatment of maternal hyperglycemia, since only women with glucose levels below those diagnostic of diabetes were included. In line with these results, the Hillier study showed an increased OR for overweight and obesity in the offspring of women with untreated GDM and no increased OR in the offspring of women with treated GDM [35]. Previous studies have shown that the combination of maternal overweight and maternal GDM has a greater impact on adverse pregnancy outcomes than either one alone [40]. In this review, we found evidence for a comparable result concerning the impact on childhood overweight. One study showed that prenatal exposure to maternal overweight combined with GDM conveyed a greater risk of childhood overweight than exposure to one of these alone [29]. Therefore, adjustment for maternal overweight may mask this potential synergistic relationship [21]. Additional adjustment for birth weight, smoking during pregnancy, prolonged breastfeeding, and socioeconomic variables did not significantly change the results. The mechanisms by which GDM might influence childhood overweight are not fully known. Fetal hyperinsulinemia during critical periods may induce leptin resistance (leptin is a hormone that reduces food intake and increases energy expenditure) [39]. Furthermore, intrauterine exposure to GDM may influence the expression of genes that direct the accumulation of body fat through epigenetic changes [21].

Of all 23 studies included in our review, only seven studies investigated the long-term risk for AGT in the offspring after GDM. Most studies confirmed that GDM was associated with an increased prevalence of AGT in the OGDM group. Only two studies showed no effect, of which one was most likely underpowered [28]. The HAPO follow-up study demonstrated an increased childhood prevalence of IGT, but not of IFG, independent of maternal BMI and childhood BMI. This finding is in line with a recent large meta-analysis [41]. It indicates that IFG and IGT may need to be considered as two distinct pathophysiologic conditions [42,43]. In addition, our review showed that GDM is associated with an increased IR and a low DI in the offspring of GDM mothers [12,14,18,34]. A low DI suggests that there is insufficient beta-cell compensation for the higher IR, and this is seen in children with a high risk to progress to T2DM [12,14].

Studies suggested that the long-term effect of GDM may not become apparent until early adolescence [44]. In this review, we confirm that the risk of childhood overweight seems higher in children >10 years, however, only two studies specifically examined different age categories.

There is limited evidence from subgroup analysis that the risk of childhood overweight and AGT is higher in female offspring compared to male offspring, but only seven studies evaluated possible sex differences. Previous research has shown that women carrying a male fetus have a 4% increased risk of developing GDM compared to women carrying a female fetus. Women developing GDM with a female fetus, therefore probably have more underlying IR and/or impaired insulin secretion, which might lead to an increased risk for long-term metabolic complications [45].

The intervention RCTs in pregnancy have shown that treatment of GDM reduces the risk for adverse pregnancy outcomes. However, follow-up studies of both RCTs showed no significant difference in the risk of childhood obesity in offspring of women who were treated for GDM compared to the untreated group. Therefore, there is currently no evidence that treatment of GDM can prevent the long-term metabolic complications in the offspring. However, the follow-up of these studies was limited to a maximum of ten years, only a subgroup of the offspring was evaluated, and the impact might also be more pronounced in offspring of mothers with more severe GDM.

### 4.3. Novelty and Practical Implications

Our review provides an updated extensive overview on the impact of GDM on the long-term metabolic risks in the offspring. In contrast to other recent reviews [46,47], we specifically assessed the impact of different confounders such as maternal BMI and the impact of age and sex of the offspring on the associated risk of GDM with overweight and AGT. Our review has several implications for clinical practice. First, we show that there is now increasing evidence that GDM is associated with an increased risk for overweight, IR, and AGT in the offspring, independent of maternal BMI. This highlights the importance to start early after delivery with follow-up in offspring of mothers with GDM to prevent and timely detect metabolic complications in this high-risk group. In addition, there is some evidence suggesting that girls have a higher risk for these long-term metabolic complications than boys. As childhood overweight is associated with a higher risk of being overweight as an adult, and an increased IR and low DI are early expressions of ATG, timely detection and treatment of GDM might reduce these long-term metabolic complications in the offspring [12]. However, larger and longer follow-up studies are needed to evaluate a potential treatment benefit. Increased awareness is needed to stimulate a sustained healthy lifestyle for the whole family starting early after delivery.

### 4.4. Strengths and Limitations

We provide an extensive narrative review on the long-term metabolic risk in offspring associated with GDM. We specifically assessed the impact of different confounders such as maternal BMI and the impact of age and sex of the offspring on the associated risk of GDM with overweight and AGT. However, our review had several limitations. We did not perform a systematic review and could not perform a meta-analysis because of the heterogeneity of studies. We did not asses the risk of bias of individual studies and did not contact the authors for obtaining missing and unpublished data. Most studies used definitions for overweight and obesity based on BMI and more detailed parameters on adiposity were often lacking. In addition, some studies corrected for current maternal BMI instead of pre-gestational maternal BMI. However, an acceptable correlation between these two BMI values has been reported [48]. Only two studies corrected for paternal diabetes, ten studies corrected for socioeconomic variables, and four studies corrected for lifestyle behaviors. Most studies investigating the impact on AGT did not correct for any confounding factor. Due to the small prevalence of T2DM in the offspring, AGT was reported instead of T2DM alone. Since the majority of included studies were cohort studies (with only two RCTs), we could not determine a causal relationship of the reported associations.

## 5. Conclusions

Our review shows that intrauterine exposure to GDM increases the risk of overweight and AGT in the offspring, independent of maternal BMI. Screening for GDM might therefore also offer a window of opportunity to prevent or reduce the risk for long-term metabolic complications in the offspring by increasing the awareness for a healthy lifestyle in this high-risk group. It remains unclear whether treatment of GDM can reduce the long-term risk of adverse metabolic complications in the offspring.

## Figures and Tables

**Table 1:** The characteristics of included studies.

Author, Year	Design	Country	Subjects (N)	Age	GDM Criteria	Comparison
Lowe, 2019 [13] (HAPO cohort)	Prospective cohort study	Multinational	4775	10–14 y	IADPSG	Continuous measures of maternal glucose levels
Lowe, 2019 [14] (HAPO cohort)	Prospective cohort study	Multinational	4775	10–14 y	IADPSG	OGDM vs. NGDM
Scholtens, 2019 [15] (HAPO cohort)	Prospective cohort study	Multinational	4160	10–14 y	IADPSG	Continuous measures of maternal glucose levels
Lowe, 2018 [16] (HAPO cohort)	Prospective cohort study	Multi-national	4832	10–14 y	IADPSG	OGDM vs. NGDM
Kaseva, 2018 [8] (ESTER and AYLS cohort)	Prospective cohort study	Finland	700	22–25 y	Finnish Diabetes Association	OGDM vs. NGDM
Le Moullec, 2018 [17] (OBEGEST cohort)	Prospective cohort study	France	1251	5–7 y	C&C	OGDM vs. NGDM
Grunnet, 2017 [18] (Danish National Birth Cohort)	Prospective cohort study	Denmark	1158	9–16 y	Self-report and the Danish National Patient Register	OGDM vs. NGDM
Tam, 2017 [19] (HAPO cohort)	Prospective cohort study	China	926	7 y	IADPSG	OGDM vs. NGDM
Bider-Canfield, 2017 [20]	Retrospective cohort study	US	15,170	2 y	C&C	OGDM vs. NGDM
Zhao, 2016 [21]	Cross-sectional	Multi-national	4740	9–11 y	WHO 1999 and ADA	OGDM vs. NGDM
Landon, 2015 [22]	Randomized controlled trial	US	500	5–10 y	C&C	Treated OGDM vs. untreated OGDM
Kelstrup, 2013 [12]	Prospective cohort study	Denmark	295	18–27 y	Local (Denmark)^*^	OGDM vs. NGDM
Nehring, 2013 [23] (German Perinatal Prevention of Obesity cohort)	Retrospective cohort study	Germany	7355	5–6 y	ADA	OGDM vs. NGDM
Regnault, 2013 [24] (Viva cohort)	Prospective cohort study	US	839	7–9 y	C&C	OGDM vs. NGDM
Pham, 2013 [25]	Retrospective cohort study	US	2093	2–4 y	Until April 2007: NDDGAfter April 2007: C&C	OGDM vs. NGDM
Patel, 2012 [26]	Prospective cohort study	Great Britain	4861	15–16 y	Questionnaire	OGDM vs. NGDM
Boerschmann, 2010 [27]	Prospective cohort study	Germany	663	2 y, 8 y, 11 y	German Diabetes Association	OGDM vs. NGDM
Tam, 2010 [28]	Prospective cohort study	China	129	15 y	ADA	OGDM vs. NGDM
Pirkola, 2010 [29] (Northern Finland Birth Cohort)	Prospective cohort study	Finland	4168	7 y, 16 y	Finnish Diabetes Association	OGDM vs. NGDM
Gillman, 2010 [30] (ACHOIS cohort)	Randomized controlled trial	Australia	199	4–5 y	Local (Australia)^**^	Routine care control group vs. intervention group
Krishnaveni, 2010 [31]	Prospective cohort study	India	416	5 y, 9 y	C&C	OGDM vs. NGDM
Lawlor, 2010 [32] (ALSPAC cohort)	Prospective cohort study	Great Britain	6516	9–11 y	Medical records	OGDM vs. NGDM
Clausen, 2009 [33]	Retrospective cohort study	Denmark	296	18–27 y	Local (Denmark)^*^	OGDM vs. NGDM
Vääräsmäki, 2009 [34] (Northern Finland Birth cohort)	Prospective cohort study	Finland	4004	16 y	Finnish Diabetes Association	OGDM vs. NGDM
Hillier, 2007 [35]	Prospective cohort study	US	8152	5–7 y	C&C and NDDG	OGDM according to C&C criteria and OGDM according to NDGG criteria vs. NGDM
Gillman, 2003 [7]	Retrospective cohort study	US	14,881	9–14 y	Interview	OGDM vs. NGDM

GDM: gestational diabetes mellitus; OGDM: offspring of mothers with gestational diabetes; NGDM: offspring of mothers with normal glucose tolerance during pregnancy; HAPO: Hyperglycemia and Adverse Pregancy Outcome; ESTER: Maternal Pregnancy Disorders and Early-Life Programming of Adult Health and Disease; AYLS: Arvo Ylppö Longitudinal Study; OBEGEST: South Reunion Island cohort; ACHOIS: Australian Carbohydrate Intolerance Study in Pregnant Women; ALSPAC: Avon Longitudinal Study of Parents and Children; IADPSG: International Association of the Diabetes and Pregnancy Study Group; C&C: Carpenter and Coustan; WHO: World Health Organization; ADA: American Diabetes Association; NDDG: National Diabetes Data Group. * Local (Denmark): Two of seven values exceeded the mean + 3SD values for a reference group of normal-weight nonpregnant women without a family history of diabetes [36]. ** Local (Australia): Fasting plasma glucose <7.8 mmoL/L (<140 mg/dL) and 2 h plasma glucose between 7.8 mmoL/L (140 mg/dL) and 11 mmoL/L (198 mg/dL) after a 2 h 75 g oral glucose tolerance test; VS: versus.

**Table 2:** The impact of GDM on overweight and adiposity in the offspring.

Article	Age	Outcome	OR for One SD Increase in Maternal Glucose Value		*p*-Value	Adjusted for
Lowe, 2019 [13]	10–14 y	Overweight or obesity ^a^				Field center, child pubertal status, maternal variables during pregnancy OGTT (age, height, any family history of diabetes, mean arterial pressure, parity, smoking, alcohol, gestational age, maternal BMI).
		FPG	1.05 (0.98, 1.14)		0.19	
		2 h glucose	1.09 (1.01, 1.17)		0.019	
		Obesity ^a^				
		FPG	1.16 (1.05, 1.29)		0.005	
		2 h glucose	1.21 (1.09, 1.34)		<0.001	
		BF% >85th percentile				
		FPG	1.15 (1.05, 1.26)		0.002	
		2 h glucose	1.15 (1.06, 1.26)		0.001	
		WC >85th percentile				
		FPG	1.09 (0.99, 1.19)		0.067	
		2 h glucose	1.17 (1.07, 1.27)		0.003	
**Article**	**Age**	**Outcome**	**OGDM**	**NGDM**	***p*-Valued**	**Adjusted for**
Lowe, 2018 [16]	10–14 y	Overweight or obesity ^a^	39.50%	28.60%		Field center, child pubertal status, maternal variables during pregnancy OGTT (age, height, any family history of diabetes, mean arterial pressure, parity, smoking, alcohol, gestational age, maternal BMI).
			1.21 (1.00, 1.46)		0.05	
		Obesity ^a^	19.10%	9.90%		
			1.58 (1.24, 2.01)		<0.001	
		BF% >85th percentile	1.35 (1.08, 1.68)		0.68	
		WC >85th percentile	1.34 (1.08, 1.67)		0.009	
Bider-Canfield, 2017 [20]	2 y	Overweight or obesity ^b^	0.96 (0.83, 1.11)		NS	Pre-pregnancy BMI, excessive gestational weight gain.
Grunnet, 2017 [18]	9–16 y	Mean difference BMI (%)	4% (2, 6)		<0.0001	Offspring age, sex, maternal pre-pregnancy BMI.
		Mean difference WC (cm)	0.52 (−0.06, 1.08)		0.08	
		Mean difference BF%	0.72% (−0.17, 1.61)		NS	
Tam, 2017 [19]	7 y	Overweight or obesity ^b^	22.70%	15.30%		Maternal age, parity, BMI before pregnancy, children’s exercise level, current maternal and paternal DM status and children’s age and/or sex.
			1.59 (0.97, 2.59)		NS	
		Obesity ^b^	8.40%	6.80%		
Zhao, 2016 [21]	9–11 y	Obesity ^c^	18.40%	12%		Child age, education, infant feeding mode, gestational age, number of younger siblings, child unhealthy diet pattern scores, moderate-to-vigorous physical activity, sleeping time, sedentary time, sex, birth weight, current maternal BMI.
			1.37 (0.92, 2.04)		0.13	
		WC ≥90th percentile	1.54 (1.01, 2.35)		0.046	
		BF% ≥90th percentile	1.30 (0.81, 2.06)		0.29	
Nehring, 2013 [23]	5–6 y	Overweight or obesity ^a^	21.00%	10.40%		Maternal pre-pregnancy BMI, Large for gestational age maternal age, gestational weight gain, breastfeeding, socio-economic status, child’s physical activity score, child’s television viewing.
			1.81 (1.23, 2.65)		<0.05	
		Obesity ^a^	8.20%	2.40%		
			2.80 (1.58, 4.99)		<0.05	
		WC ≥90th percentile	1.64 (1.16, 2.33)		<0.05	
Pham, 2013 [25]	2–4 y	Overweight or obesity ^b^	23.90%	23.50%		Maternal age, height, race or ethnicity, child age.
			0.9 (0.7, 1.3)		NS	
Patel, 2012 [26]	15–16 y	Overweight or obesity ^a^	29.60%	16.40%		Sex, age, maternal age, manual social class, maternal smoking during pregnancy, parity, maternal pre-pregnancy BMI, gestational age, birth weight, mode of delivery.
			0.54 (0.10, 3.03)		NS	
		WC 90th percentile	0.90 (0.32, 2.52)		NS	
Lawlor, 2010 [32]	9–11 y	Overweight or obesity ^a^	30%	23%		Sex, age, gestational age, height and height squared in models with fat mass as outcome, maternal age, social class, parity, smoking during pregnancy, mode of delivery, maternal pre-pregnancy BMI.
			0.62 (0.32, 1.23)		NS	
		WC ≥90th percentile	48%	38%		
			1.00 (0.55, 1.85)		NS	
Pirkola, 2010 [29]	16 y	Overweight or obesity ^a^	Overweight mother: 4.05 (1.09, 8.62)		<0.001	Maternal overweight, maternal smoking status, paternal overweight, paternal smoking status, sex, birth weight.
			Normal weight mother: 0.73 (0.26, 2.08)		NS	
Clausen, 2009 [33]	18–27 y	Overweight or obesity ^d^	40%	24%		Maternal age at delivery, maternal pregestational BMI, offspring age, family occupational social class, maternal hypertension at first visit.
			1.79 (1.00, 3.24)		<0.05	
Vääräsmäki, 2009 [34]	16 y	Overweight ^d^	18.80%	8.40%		Birth weight, gestational age and sex.
		Obesity ^e^	6.40%	1.90%		
		WC ≥94 cm in men and	3.10 (1.28, 7.52)		<0.05	
		≥80 cm in women	2.71 (1.52, 4.82)		<0.05	
Hillier, 2007 [35]	5–7 y	C&C				Maternal age, parity, weight gain during pregnancy, ethnicity, macrosomia at birth (>4.000 g), sex.
		Overweight ^b^	34.70%	23.50%		
			1.89 (1.30, 2.76)		<0.05	
		Obesity^b^	20.20%	12.20%		
			1.82 (1.15, 2.88)		<0.05	
		NDDG				
		Overweight ^b^	27.80%	23.50%		
			1.29 (0.85, 1.97)		NS	
		Obesity ^b^	17.30%	12.20%		
			1.38 (0.84, 2.27)		NS	
Gillman, 2003 [7]	9–14 y	Overweight ^b^	17.10%	14.20%		Age, gender, tanner stage, television watching, physical activity, energy intake, breastfeeding duration, birth order, household income, mother’s smoking, dietary restraint, weight cycling, weight concerns, birth weight, mother’s current BMI.
			1.0 (0.7, 1.3)		NS	
		Obesity ^b^	9.70%	6.60%		
			1.2 (0.8, 1.7)		NS	

Data are expressed as prevalence (%), odds ratio or mean differences (SD). We only mentioned the most adjusted data of each study. OGDM: offspring of mothers with gestational diabetes; NGDM: offspring of mothers with normal glucose tolerance during pregnancy; FPG: fasting plasma glucose; 2 h glucose: maternal glucose values 2 h after a 75 g OGTT; BF%: Body Fat Percentage; WC: waist circumference; BMI: Body Mass Index; C&C: Carpenter and Coustan; NDDG: National Diabetes Data Group; NS: not significant; OGTT: oral glucose tolerance test. a: According to sex- and age specific cut-offs based on the International Obesity Task Force. b: According to sex- and age specific BMI percentiles based on the Centers of Disease Control and Prevention. c: According to sex- and age specific BMI z-score based on the WHO growth reference. d: BMI ≥ 25 kg/m^2^. e: BMI ≥ 30 kg/m^2^. f: BMI ≥ 90th percentile adjusted for age and sex according to German reference data.

**Table 3:** The impact of GDM on glucose intolerance and insulin resistance in the offspring.

Article	Age	Outcome	OGDM	NGDM	*p*-Value	Adjusted for
Lowe, 2019 [14]	10–14 y	FPG (mmoL/L)	5.1 (4.7, 5.5)	5.0 (4.6, 5.4)	NS	Field center, child age, child sex, pubertal status, maternal variables at pregnancy OGTT (age, height, mean arterial pressure, parity, smoking, drinking, gestational age), child’s family history of diabetes in first-degree relatives, maternal BMI at pregnancy OGTT, child’s BMI z-score.
		IFG ^a^	9.20%	7.40%		
			1.09 (0.78, 1.52)		0.61	
		IGT ^a^	10.60%	5.00%		
			1.96 (1.41, 2.73)		<0.001	
		RC Matsuda index ^d^	−76.3 (−130.3, −22.4)		0.0063	
		RC Insulinogenic index ^f^	−0.06 (−0.12, 0.003)		0.061	
		RC Disposition index ^g^	−0.12 (−0.17, −0.064)		<0.0001	
Grunnet, 2017 [18]	9–16 y	FPG (mmoL/L)	5.0 (4.2, 5.8)	4.8 (4.2, 5.4)	<0.001	Age, sex, offspring BMI, maternal pre-pregnancy BMI.
		Mean difference FPG (%)	4% (2, 5)			
		HOMA-IR ^h^	2.2 (0.6, 3.8)	1.9 (0.8, 3)	0.02	
		Mean difference HOMA-IR (%)	8% (1, 16%)			
Tam, 2017 [19]	7 y	FPG (mmoL/L)	4.57 (4.22, 4.92)	4.64 (4.15, 5.13)	0.12	No adjustments made
		IFG and/or IGT ^a^	3.90%	1.70%	0.04	
		DM type II ^a^	0.80%	0%	0.04	
		Matsuda index ^c^	15.0 (6.7, 23.3)	16.2 (7.3, 25.1)	0.14	
		Insulinogenic index ^e^	67.8 (2.8, 132.8)	81 (−13.2, 175.2)	0.05	
		Oral disposition index ^i^	6.6 (0.7, 12.6)	7.9 (−1.5, 17.4)	0.04	
Kelstrup, 2013 [12]	18–27 y	IFG ^b^ and/or IGT ^b^ and/or DM type II ^b^	21%	4%	<0.0001	No adjustments made
		HOMA-IR ^h^	10.53 (9.58, 11.57)	8.47 (7.71, 9.31)	<0.05	
		Insulinogenic index ^e^	86.9 (76.6, 96.4)	90.3 (80.1, 101.9)	NS	
		Disposition index ^j^	15,743 (13877, 17861)	24,820 (22197, 27752)	<0.05	
Tam, 2010 [28]	15 y	FPG (mmoL/L)	4.6 (4.3, 4.9)	4.7 (4.4, 5.0)	0.51	No adjustments made
		IFG ^a^ and/or IGT ^a^ and/or DM type II ^a^	11.90%	10.30%	0.77	
Vääräsmaki, 2009 [34]	16 y	FPG (mmoL/L)	5.30 (5.00, 5.50)	5.10 (4.90, 5.40)	NS	Birth weight, gestational age, sex, current BMI.
		IFG ^a^ and/or IGT ^a^ and/or DM type II ^a^	23.60%	15.30%		
			1.63 (0.97, 2.74)		NS	
		HOMA-S ^h^	74.7 (54.1, 91.2)	82.3 (64.0, 104.7)	<0.05	
Clausen, 2009 [33]	18–27 y	IFG ^a^ and/or IGT ^a^ and/or DM type II ^a^	41%	10%	<0.05	No adjustments made

The outcomes “Impaired Fasting Glucose”, “Impaired Glucose Tolerance” and “Diabetes Mellitus Type II” are expressed as prevalence (%) or odds ratio’s. The other data are mean (SD), unless specified otherwise. OGDM: offspring of mothers with gestational diabetes; NGDM: offspring of mothers with normal glucose tolerance during pregnancy; FPG: Fasting Plasma Glucose; OGTT: oral glucose tolerance test; IFG: Impaired Fasting Glucose; IGT: Impaired Glucose Tolerance; RC: Regression Coefficient; HOMA-IR: Homeostatic Model Assessment of Insulin Resistance; DM type II: Diabetes Mellitus type II; HOMA-S: Homeostatic Model Assessment of Insulin Sensitivity; NS: not significant. a: According to the American Diabetes Association diagnostic criteria. b: According to the World Health Organization criteria of 1999. c: According to the formula described by Matsuda [9]. d: Modified Matsuda index [14]. e: According to the formula described by Phillips [11]. f: Modified Insulinogenic index [14]. g: Log transformed: Matsuda index x insulinogenic index [14]. h: According to the formula described by Matthews [10]. i: Insulinogenic index x Matsuda index [19]. j: Corrected insulin response x Matsuda index [12].

## Appendix A

**Table A1:** This list gives an overview of the most commonly used gestational diabetes mellitus diagnosis criteria.

Criteria	OGTT	FPG	1 h	2 h	3 h	Number of Abnormal Values
C&C	100 g	≥5.3 mmoL/L (=95 mg/dL)	≥10 mmoL/L (=180 mg/dL)	≥8.6 mmoL/L (=155 mg/dL)	≥7.8 mmoL/L (=140 mg/dL)	≥2
NDDG	100 g	≥5.8 mmoL/L (=105 mg/dL)	≥10.5 mmoL/L (=190 mg/dL)	≥9 mmoL/L (=165 mg/dL)	≥8 mmoL/L (=145 mg/dL)	≥2
IADPSG, WHO 2013	75 g	≥5.1 mmoL/L (=92 mg/dL)	≥10 mmoL/L (=180 mg/dL)	≥8.5 mmoL/L (=153 mg/dL)		≥1
ADA	100 g	≥5.3 mmoL/L (=95 mg/dL)	≥10 mmoL/L (=180 mg/dL)	≥8.6 mmoL/L (=155 mg/dL)	≥7.8 mmoL/L (=140 mg/dL)	≥2
WHO 1999	75 g	≥7 mmoL/L (=126 mg/dL)		≥7.8 mmoL/L (=140 mg/dL)		≥1
German Diabetes Association	75 g	>5 mmoL/L (=90 mg/dL)	>10 mmoL/L (=180 mg/dL)	>8.6 mmoL/L (=155 mg/dL)		≥2
Finnish Diabetes Association	75 g	>5.5 mmoL/L (=99 mg/dL)	>11.0 mmoL/L (=198 mg/dL)	>8.0 mmoL/L (=144 mg/dL)		≥1

OGTT: oral glucose tolerance test; FPG: fasting plasma glucose; C&C: Carpenter and Coustan; NDDG: National Diabetes Data Group; IADPSG: International Association of the Diabetes and Pregnancy Study Groups; ADA: American Diabetes Association; WHO: World Health Organization.
